# Paradoxical reaction to midazolam reversed with flumazenil

**DOI:** 10.4103/0974-2700.66551

**Published:** 2010

**Authors:** Luciano Santana Cabrera, Ana Sánchez Santana, Pilar Eugenio Robaina, Manuel Sánchez Palacios

**Affiliations:** Intensive Care Unit, Universitary Hospital Insular in Gran Canaria, Las Palmas de Gran Canaria, Spain

Sir,

Midazolam belongs to a class of medications called benzodiazepines and it is used before medical procedures or before anesthesia for surgery to cause drowsiness and sleep, relieve anxiety, and prevent any memory of event. Midazolam may cause side-effects, some of which may be serious, such as agitation, restlessness, uncontrollable shaking of a part of the body, stiffening and jerking of the arms and legs, and aggression. This paradoxical excitement occurs in <1% of all patients receiving midazolam, which may be reversed with flumazenil, avoiding increasing the level of sedation.[1,2] In this report, we describe a patient who developed a paradoxical reaction to midazolam that was reversed with flumazenil.

A 38-year-old man who had no previous history of psychotic or neurotic disorders occasionally used cocaine and did not use narcotics. He was previously intervened with colonic diverticulitis using the technique of Hartman, and was now admitted to carrying out reconstruction of the intestinal transit. After surgery, he was consistently awake, aware, calm and without focal neurologic changes until he suffered from respiratory depression by opiates, used as analgesic medication, and, therefore, was reintubated with 15 mg midazolam after the procedure. After 2 h, the patient did not respond to requests to open his eyes. The Glasgow Coma Score was 8–10 points and he had significant psychomotor agitation, without neurological focus, which did not lessen even after the administration of haloperidol and dipotassium clorazepate. During that time, we excluded other causes of agitation because the vital signs remained stable and hypoxia, hypercapnia, hypertension, and increased intracranial pressure could be excluded. He did not complain of any pain or discomfort, hypoglycemia was ruled out, and the electrolyte imbalance did not exist to justify the patient’s symptoms. It is decided to administer flumazenil 0.5 mg intravenously, immediately after which the patient became conscious, oriented and calm, the paradoxical reaction was terminated, and he had no recollection of the events. Computed tomography of the brain was not performed because the patient was fully awake after the administration of flumazenil.

Benzodiazepines stimulate the effect of gamma-aminobutyric acid (GABA) in the ascending reticular activating system. Paradoxical reactions have been described for many drugs that interact with the GABA receptor,[3] including barbiturates, volatile anesthetics, and etomidate. There are different theories concerning the mechanism of paradoxical reactions, involving a central cholinergic effect or the serotonin imbalance, although the exact mechanism of paradoxical reactions remains unclear. Most cases are idiosyncratic; however, some evidence suggests that these reactions may occur secondary to a genetic link, history of alcohol abuse, or psychological disturbances.[4,5] Midazolam is used usually by us as recommended by the clinical practice guideline based on the evidence for the management of sedoanalgesia in the critically ill adult patient[6] (Recommendation 1B).

Flumazenil is a benzodiazepine antagonist that cannot just reverse sedation, hypnosis, and respiratory depression induced by the benzodiazepine, but several reports have also demonstrated its role in the successful treatment of benzodiazepine-induced paradoxical reactions.[3] The onset of flumazenil’s effect is seen at 1–3 min, and its peak effect occurs at 6 min. Flumazenil should be used cautiously if the patient has a history of seizures controlled with benzodiazepines. Monitoring the patient post-operatively is essential because resedation might occur and the paradoxical reaction might reappear, although this has not been reported. If the patient experiences the clinical signs again, flumazenil administration should be repeated, assessing the need to administer a continuous infusion of flumazenil.

Although delirium caused by midazolam or propofol in different patients has been reported, there are cases of delirium caused by midazolam alone and by propofol in the same patient.[7]

In conclusion, this case report demonstrates that paradoxical reactions to midazolam can be treated with small doses of flumazenil. Moreover, saying that since the sedoanalgesia is an integral part of the management of critical patients in intensive care units and emergency services, is essential to know the clinical guidelines for evidence-based management of these drugs.[6]
